# The LRRK2 G2385R variant is a partial loss-of-function mutation that affects synaptic vesicle trafficking through altered protein interactions

**DOI:** 10.1038/s41598-017-05760-9

**Published:** 2017-07-14

**Authors:** Maria Dolores Perez Carrion, Silvia Marsicano, Federica Daniele, Antonella Marte, Francesca Pischedda, Eliana Di Cairano, Ester Piovesana, Felix von Zweydorf, Elisabeth Kremmer, Christian Johannes Gloeckner, Franco Onofri, Carla Perego, Giovanni Piccoli

**Affiliations:** 1 CIBIO, Università degli Studi di Trento, Italy & Dulbecco Telethon Institute, Trento, Italy; 2 Dept of Pharmacological and Biomolecular Sciences Università degli Studi di Milano, Milano, Italy; 3 Department of Experimental Medicine, University of Genova, Genova, Italy; 4 Institute of Molecular Immunology, Helmholtz-Zentrum München, Munich, Germany; 5 German Center for Neurodegenerative Diseases (DZNE), Tübingen, Germany; 6 Eberhard Karls University, Institute for Ophthalmic Research, Center for Ophthalmology, Tübingen, Germany

## Abstract

Mutations in the Leucine-rich repeat kinase 2 gene (*LRRK2*) are associated with familial Parkinson’s disease (PD). LRRK2 protein contains several functional domains, including protein-protein interaction domains at its N- and C-termini. In this study, we analyzed the functional features attributed to LRRK2 by its N- and C-terminal domains. We combined TIRF microscopy and synaptopHluorin assay to visualize synaptic vesicle trafficking. We found that N- and C-terminal domains have opposite impact on synaptic vesicle dynamics. Biochemical analysis demonstrated that different proteins are bound at the two extremities, namely β3-Cav2.1 at N-terminus part and β-Actin and Synapsin I at C-terminus domain. A sequence variant (G2385R) harboured within the C-terminal WD40 domain increases the risk for PD. Complementary biochemical and imaging approaches revealed that the G2385R variant alters strength and quality of LRRK2 interactions and increases fusion of synaptic vesicles. Our data suggest that the G2385R variant behaves like a loss-of-function mutation that mimics activity-driven events. Impaired scaffolding capabilities of mutant LRRK2 resulting in perturbed vesicular trafficking may arise as a common pathophysiological denominator through which different LRRK2 pathological mutations cause disease.

## Introduction

Parkinson’s disease (PD) is the second most common age-related neurodegenerative disease clinically characterized by movement impairments such as bradykinesia, rigidity and resting tremor and pathologically by the progressive death of dopaminergic neurons in the *substantia nigra* and the deposition of protein aggregates known as Lewy bodies^1,2^. Although the majority of cases are idiopathic, mutations in the Leucine-rich repeat kinase 2 *(LRRK2)* gene (PARK8; OMIM 609007) have been robustly correlated to late-onset autosomal dominant PD. LRRK2 mutations are responsible for up to 13% of familial PD cases and have been identified also in 1 to 2% of idiopathic PD patients^3^. LRRK2 is a large protein with a dual enzymatic activity and two domains involved in protein-protein interaction^4^. Our previous studies suggest that LRRK2 acts at the synaptic site where it binds to synaptic vesicles (SV)^5,6^. In addition, LRRK2 rodent models show neurotransmission defects^7–9^ and LRRK2 binds and phosphorylates several presynaptic proteins^10,11^. Since its initial description as PD gene, a major focus has been dedicated to LRRK2’s GTPase and the kinase domain, linking pathological mutations to altered enzymatic activities^12^. Robust evidences show that kinase activity influences LRRK2 dimerization^13,14^, subcellular distribution^15^ and regulates binding to 14-3-3 proteins^16,17^. Accordingly, our previous work suggests that LRRK2 kinase activity modulates its supra-molecular organization and ultimately LRRK2 function at the synaptic site^5^. LRRK2 N-terminal Armadillo, LRR and Ankyrin repeats as well as the C-terminal WD40 domain have been predicted to be involved in protein interactions^18,19^. In this study, we demonstrate that LRRK2 interacts with different partners that exert divergent functions in SV trafficking. Importantly, strong genetic association suggests that the missense substitution of glycine 2385 to arginine (G2385R) within the C-terminal WD40 domain is a pathologically relevant variant. The G2385R variant is associated with an increased risk of developing idiopathic PD in Chinese Han and Korean ethnicity^20–22^. We show here that the G2385R variant alters the LRRK2-associated protein interactome and reduces its binding to SV. By combining TIRF microscopy to synaptopHluorin detection, we noticed that LRRK2-G2385R expression as well as LRRK2 silencing resulted in an increase in SV fusion events, suggesting that the G2385R variant is a partial loss-of-function mutation.

## Results

### Domain-wise dissection of LRRK2 impact on vesicle trafficking

We contributed to accumulating literature describing the role played by LRRK2 in regulating SV fusion events^5,6,10,23^. To further explore the functional role of LRRK2, we combined synaptopHluorin assay to TIRF microscopy (TIRFM) in the neuroblastoma cell line SH-SY5Y.

**Figure 1:**
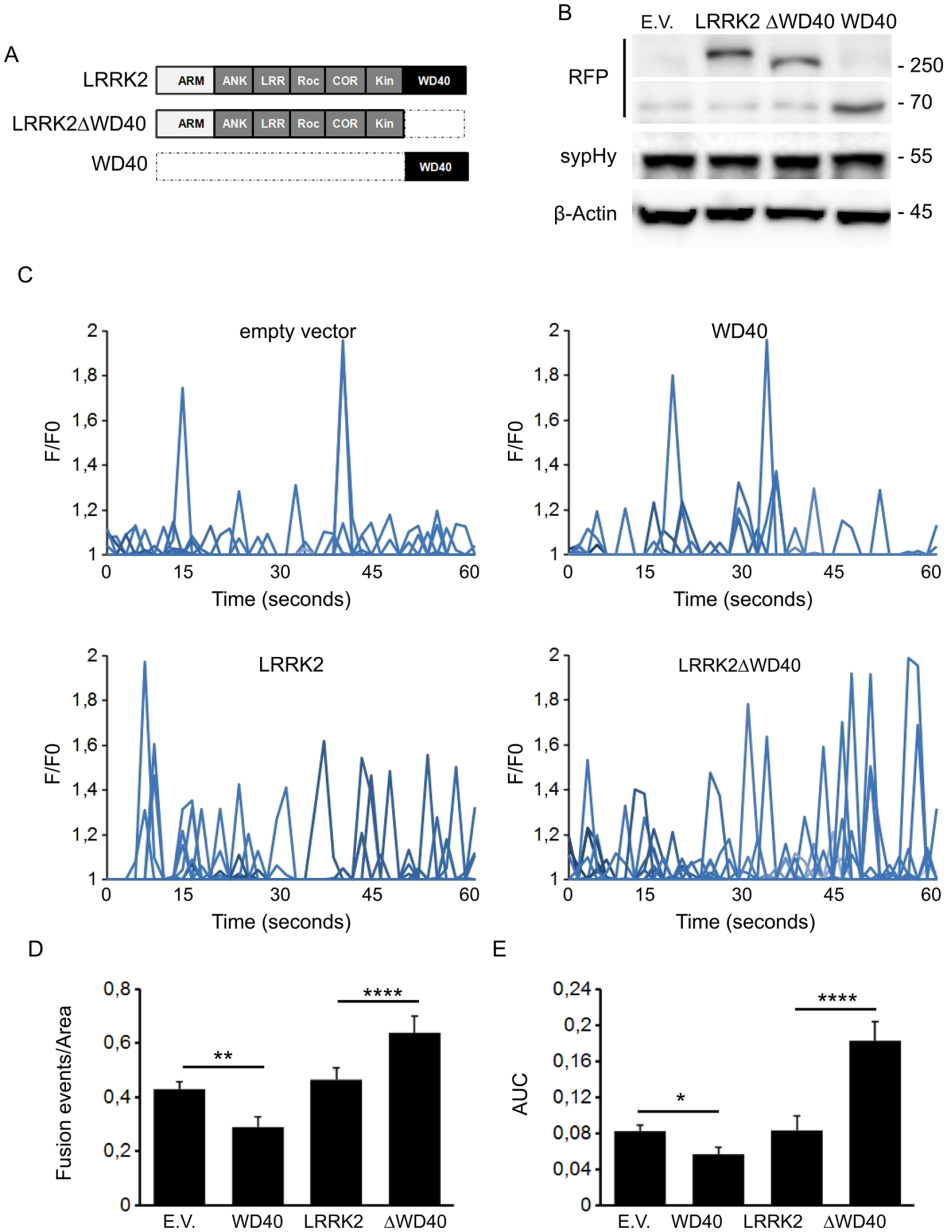
Domain-wise dissection of LRRK2 impact on vesicle trafficking. (**A**) Schematic representation of RFP-LRRK2 derived constructs. The distinct LRRK2 domains are indicated. Protein-protein domains: ARM, armadillo repeats; ANK, ankyrin repeats; LRRs, leucine-rich repeats, WD40, WD40 repeats; Roc, Ras of complex proteins; COR, C-terminal of ROC, Kin: kinase domain. (**B**) Western-blotting analysis of cells expressing synaptopHluorin reporter (sypHy) together with RFP-LRRK2 derived constructs or empty vector (E.V.). (**C**) Time course analysis of fusion events occurring in SH-5YSY cells transfected with the different LRRK2 derived constructs. SH-5YSY cells were co-transfected with sypHy reporter and empty vector (E.V.) or the indicated RFP-LRRK2 derived constructs. TIRFM imaging was performed 48 hours after transfection. Peaks of fluorescence intensity correspond to single fusion events. Fluorescence data are expressed as F/F0. The graphs show the total number of fusion events (**D**) and the resulting fluorescence changes (**E**) expressed as Area Under Curve (AUC) for each construct. Data are normalized for the cell area and are expressed as mean ± SE; n = 20 cells per construct. *p < 0.05, **p < 0.01, ****p < 0.001 ANOVA.

SynaptopHluorin (sypHy) is a fluorescent reporter of vesicle fusion and recycling. It consists of a pH-sensitive GFP fused to the luminal side of the synaptophysin protein^24^. At the acidic pH inside transmitter vesicles, sypHy fluorescence is low. Upon vesicle release, sypHy is exposed to the neutral extracellular space and fluorescence abruptly increases. We used the sypHy assay with the SH-SY5Y line which expresses LRRK2 together with a panel of SV- associated proteins. We determined that this line shuffles synapto-pHluorin reporter (sypHy) to VAMP2 positive vesicles (Supplementary Figure 1A,B) and exposes sypHy on the membrane as a consequence of SV fusion events^25^. Consequently, we over-expressed a panel of LRRK2 derived expression constructs together with sypHy reporter in the SH-SY5Y line (Fig. 1A). Western-blotting analysis showed that LRRK2-derived constructs are expressed in a similar extent and do not significantly affect sypHy level compared to control empty vector transfected cells (Fig. 1B and Supplementary Figure 1C,D). Next we analysed SV dynamics by TIRFM. Upon over-expression of full-length LRRK2 we did not notice any major impact on SV dynamics in terms of number of events, total fluorescence elicited (Fig. 1C–E) or increase in peak fluorescence intensity (Supplementary Figure 1E). Our previous data showed that the C-terminal LRRK2 WD40 domain has a major role in controlling SV trafficking^6^. Therefore we investigated SV trafficking upon over-expression of the isolated LRRK2 WD40 domain. According to our previous data, we found that LRRK2 WD40 domain does not influence single peak intensity (Fig. 1C and Supplementary Figure 1E) but severely reduces the number of fusion events (Fig. 1D,E). To further characterize the functional role of LRRK2 WD40 domain, we studied SV trafficking upon expression of LRRK2 construct lacking the WD40 domain (hereinafter LRRK2ΔWD40). Interestingly we noticed that the expression of LRRK2ΔWD40 increased the frequency of fusion events (Fig. 1C–E). All together, these findings suggest that the LRRK2 N-terminal fragment and WD40 domain have opposite impact on SV dynamics.

### LRRK2 interactome is domain specific and modulated by activity

**Figure 2:**
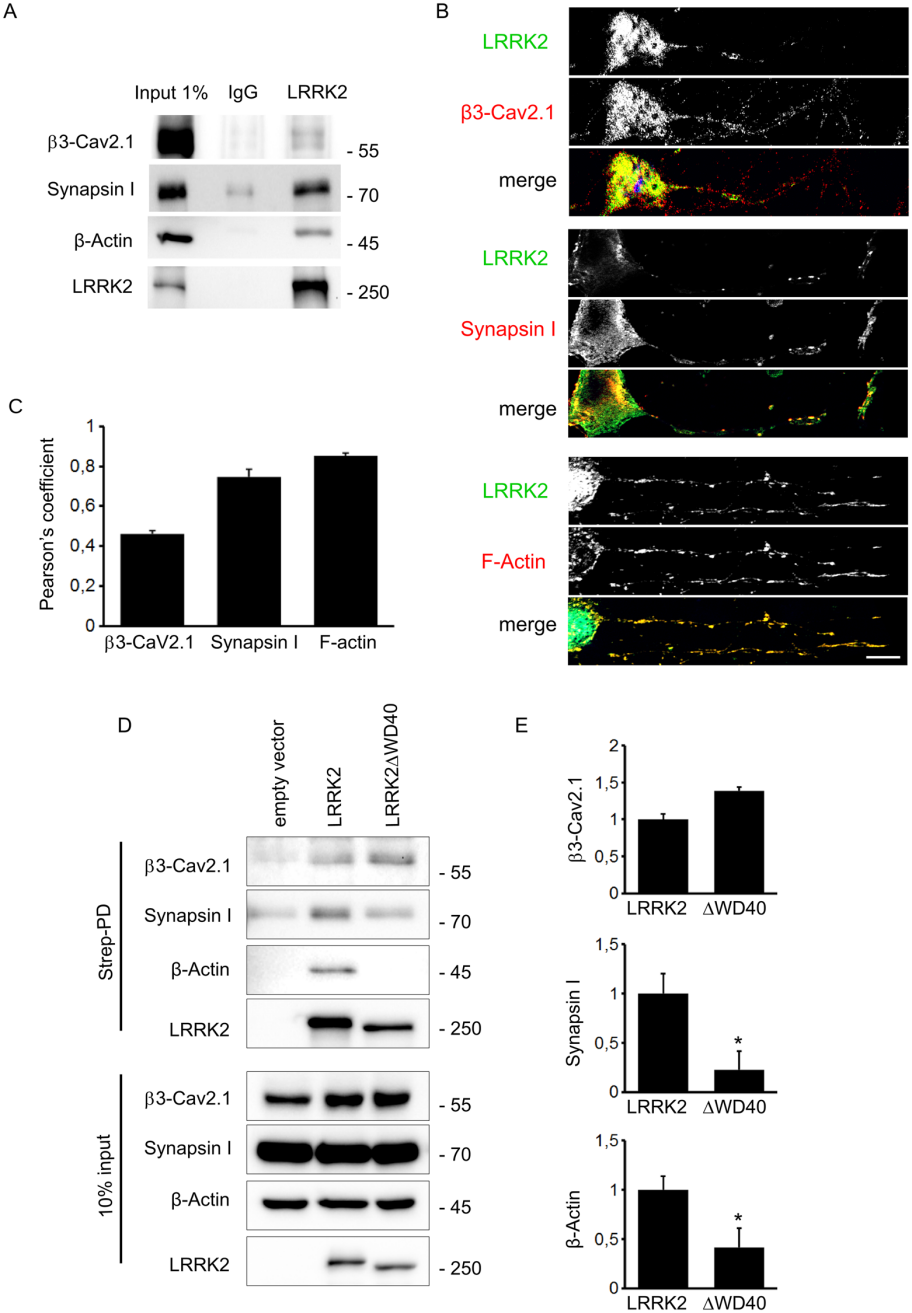
LRRK2 interacts with proteins involved in vesicle trafficking. (**A**) Immunoprecipitation of endogenous LRRK2 from adult forebrain mouse lysate shows that LRRK2 interacts with β3-Cav2.1, synapsin I and β-Actin. (**B**) DIV14 cortical neurons were processed for imaging purposes and stained with specific antibodies recognizing LRRK2, β3-Cav2.1, Synapsin I and F-Actin. (**C**) Graph shows the Pearson’s correlation coefficient to appreciate the overlap between LRRK2 and the indicated protein. (**D**) We isolated on streptavidin resin full-length strep-FLAG-LRRK2 and strep-FLAG-LRRK2ΔWD40 protein from N2A over-expressing cells. Interacting proteins were resolved by western-blotting. (**E**) We evaluated the extent of β3-Cav2.1, Synapsin I and β-Actin bound to the different LRRK2 variants. Data are expressed as optical density and normalized versus amount of precipitated LRRK2 protein. Graphs report mean ± S.E, *p < 0.05, Student’s T-test, n = 4.

**Figure 3:**
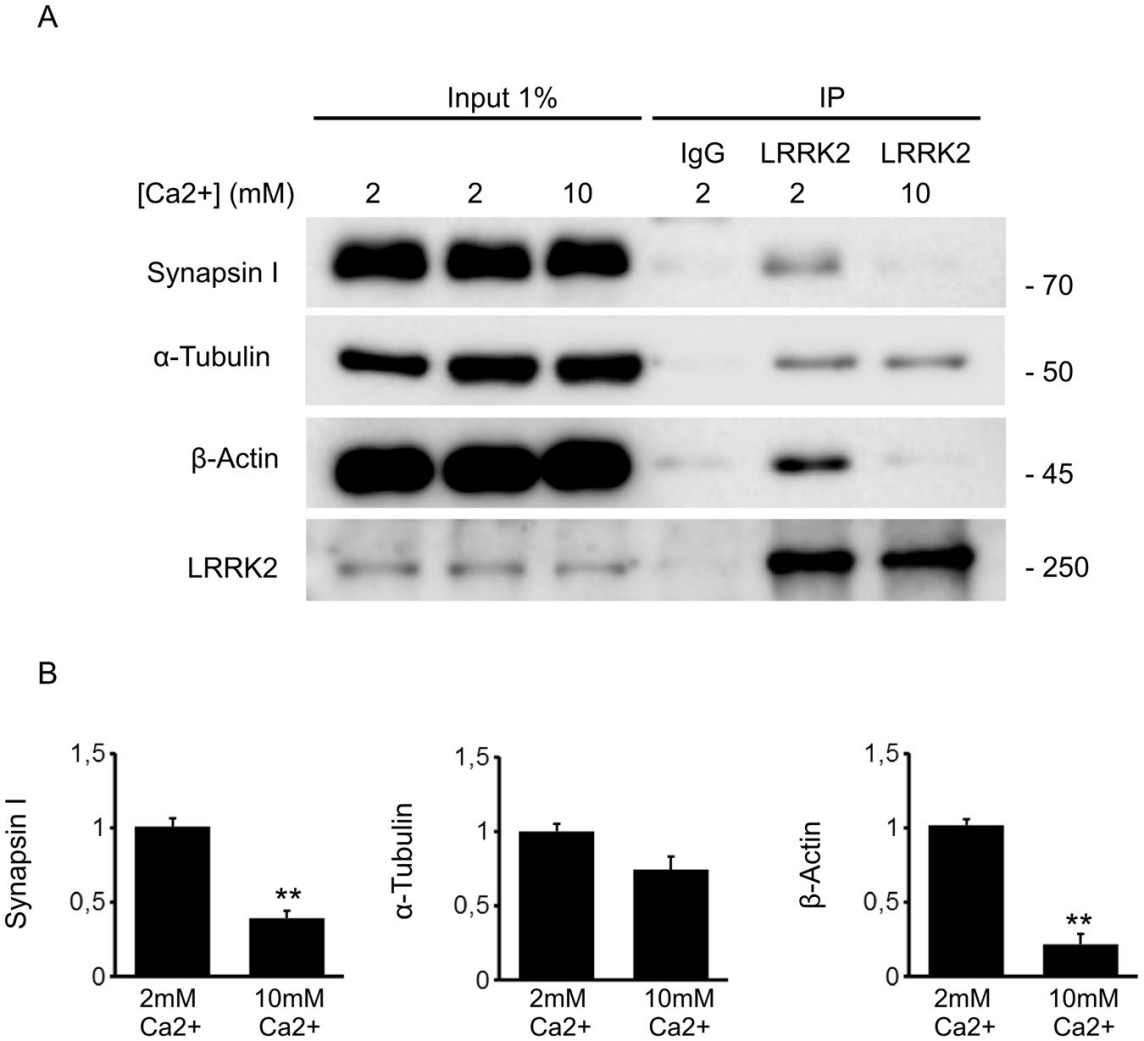
LRRK2 interactome is shaped by neuronal activity. (**A**) Immunoprecipitation of endogenous LRRK2 from DIV14 cortical culture incubated with 2 or 10 mM CaCl2 for 30 minutes. (**B**) We evaluated the extent of synapsin I, α-tubulin and β-actin bound to LRRK2 at 2 and 10 mM CaCl_2_. Data are expressed as optical density and normalized versus amount of precipitated LRRK2 protein. Graphs report mean ± S.E, **p < 0.01, Student’s T-test, n = 4.

Our previous data suggested that LRRK2 influences SV trafficking via binding to presynaptic proteins^5,6,10,23^. In particular, immunoprecipitation of endogenous LRRK2 protein from mouse forebrain lysates showed that LRRK2 interacts with β3-Ca_V_ 2.1 channel subunit, synapsin I and β-actin (Fig. 2A). To better characterize actin, synapsin and β3 Ca_V_ 2.1 channel subunit as LRRK2 interactors, we evaluated their sub-cellular localization in cortical neurons. To this aim, DIV14 cortical neurons were fixed and stained with LRRK2, F-actin, synapsin and β3-Ca_V_ 2.1 channel subunit specific antibodies (Fig. 2B). Interestingly, the three proteins showed a high degree of colocalization with LRRK2 as judged by Pearson’s coefficient (Fig. 2C). LRRK2 harbours at least two major sites for protein interaction featured by its N- and C-terminus. Thus, we mapped the LRRK2 domain hosting the interaction with β3-Ca_V_ 2.1 channel subunit, synapsin I and β–actin. To this aim, we expressed Strep-FLAG LRRK2 full-length and Strep-FLAG LRRK2ΔWD40 in N2A cells. Upon streptavidin-pull-down, we studied interacting proteins by western-blotting (Fig. 2D). Interestingly, we noticed that while the β3-Ca_V_ 2.1 channel subunit was equally retained by full-length LRRK2 and LRRK2ΔWD40, the interaction with synapsin I and β–actin was severely reduced in the LRRK2ΔWD40 variant (Fig. 2E). In conclusion, these data suggest that LRRK2 binds the β3-Ca_V_ 2.1 channel subunit via its N-terminus while β–actin and synapsin I binding is mainly mediated by the C-terminal WD40 domain. SV move along tubulin tracks, are tethered by actin and synapsin in a resting state and are released upon presynaptic increase of local [Ca^2+^]^26,27^. Thus we triggered exocytosis by increasing bath [Ca^2+^] to 10 mM for 30 minutes in DIV14 cortical cultures^28^ and we investigated LRRK2 interacting proteins via immunoprecipitation. We noticed that LRRK2 interaction with β–actin and synapsin I was robustly reduced upon stimulation (Fig. 3A,B). This evidence suggests that LRRK2 interactome is shaped by neuronal activity.

### The G2385R PD-risk variant is a partial loss-of-function mutation

**Figure 4:**
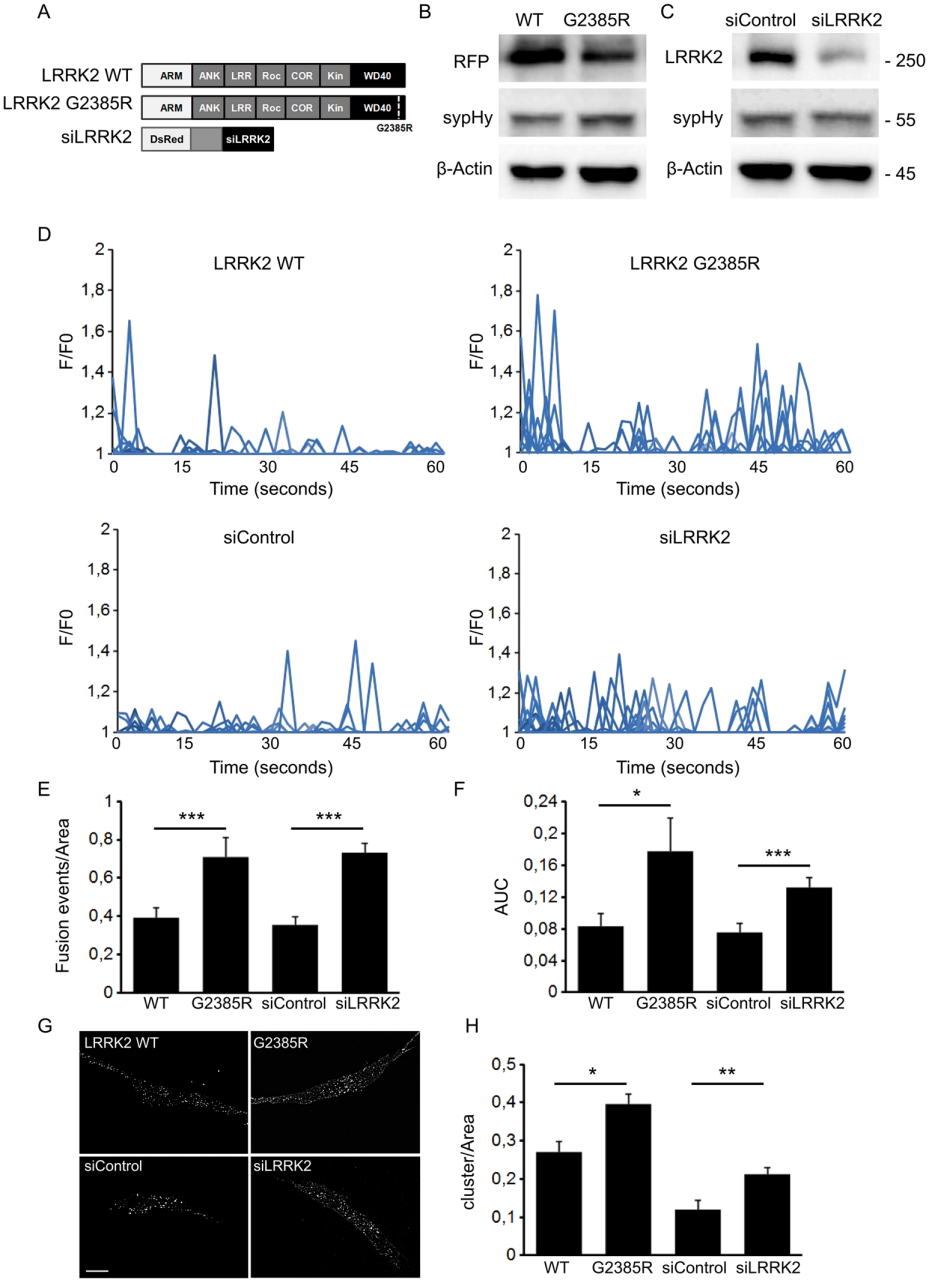
G2385R mutation affects vesicle trafficking. (**A**) Schematic representation of LRRK2 wild-type and G2385R variant. The distinct LRRK2 domains are indicated. (**B**) Western-blotting analysis of cells expressing sypHy reporter together with RFP-LRRK2 derived constructs. (**C**) Western-blotting analysis of cells expressing sypHy reporter together with vector coding for DsRed fluorescent protein and shRNA targeting hLRRK2 (siLRRK2) or a control sequence (siControl). (**D**) Time course analysis of synaptic events occurring in SH-5YSY cells transfected with wild-type LRRK2 (WT), G2385R-LRRK2, siControl or siLRRK2 under resting conditions. SH-5YSY cells were co-transfected with sypHy and the indicated constructs and imaged by TIRFM 48 hours later. Peaks of variable fluorescence intensity correspond to single fusion events. Fluorescence data are expressed as F/F0. The graphs show the total number of fusion events (**E**) and the resulting fluorescence changes (**F**) expressed as Area Under Curve (AUC) for each construct. Data are normalized for the cell area and are the mean ± SE of up to 20 cells per construct. *p < 0.05, ***p < 0.001 ANOVA. (**G**) Vesicle density in the TIRFM zone after NH_4_Cl_2_ treatment. The cells were incubated with the membrane permeant NH_4_Cl_2_ solution for 5 minutes, to label all sypHy positive cluster. Then, cells were fixed and imaged by TIRFM to visualize vesicles docked to the plasma membrane. Each spot corresponds to a sypHy positive clusters. Scale bar = 10 μm. (**H**) The graph reports the number of sypHy positive clusters present under the TIRF zone. Data are normalized for the cell area and are expressed as mean ± SE; n = 15 cells for construct. *p < 0.05, **p < 0.01 ANOVA.

**Figure 5:**
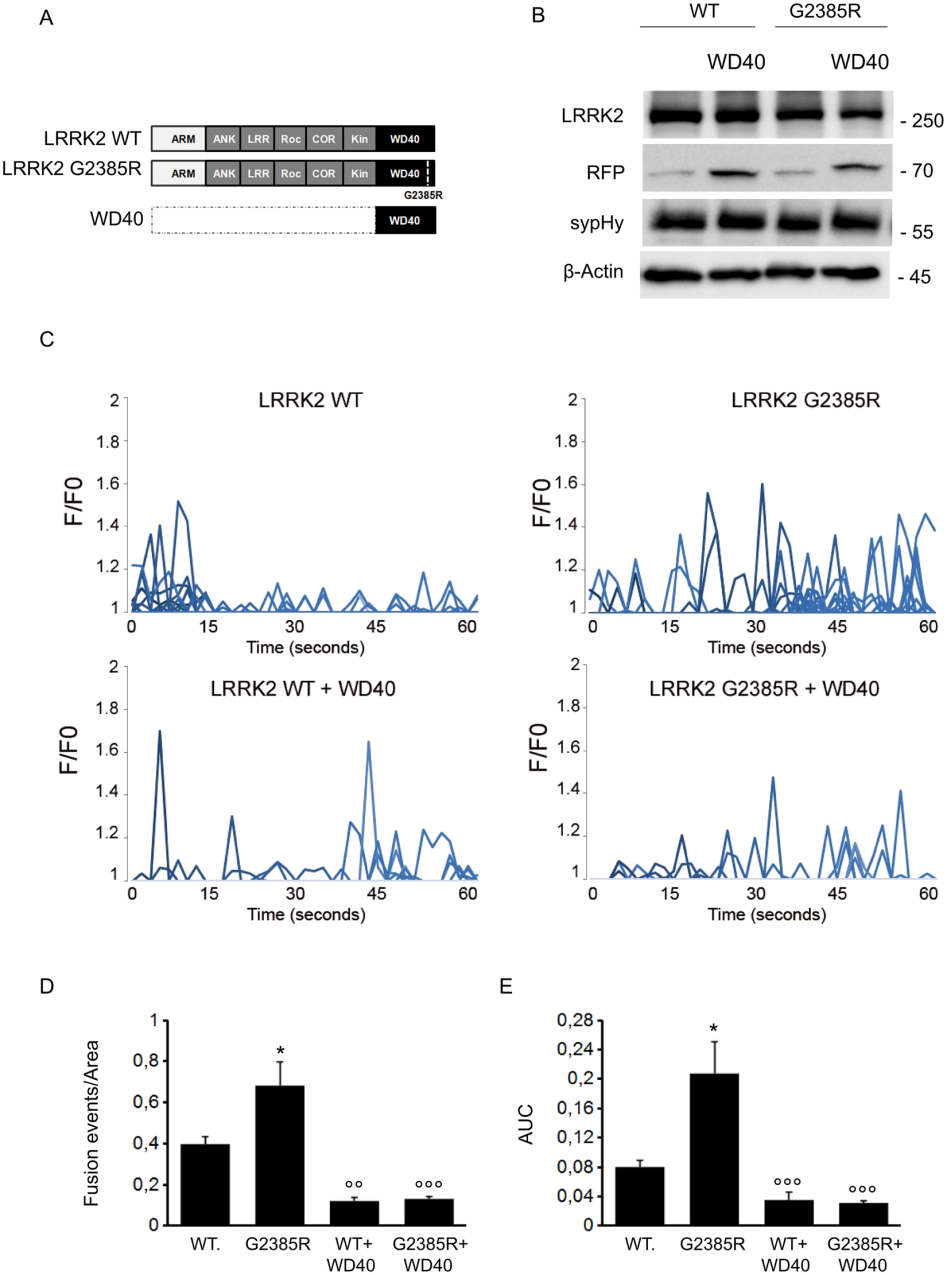
WD40 expression rescues G2385R impact on vesicle trafficking. (**A**) Schematic representation of LRRK2 wild-type and G2385R variant and isolated WD40 domain. The distinct LRRK2 domains are indicated. (**B**) Western-blotting analysis of cells expressing sypHy reporter together with RFP-LRRK2 derived constructs. (**C**) Time course analysis of fusion events occurring in stable N2A line expressing wild-type LRRK2 (WT) or G2385R-LRRK2 and transfected with sypHy together with empty vector or RFP-WD40. N2A cells were co-transfected with sypHy and the indicated constructs and imaged by TIRFM 48 hours later. Peaks of variable fluorescence intensity correspond to single fusion events. Fluorescence data are expressed as F/F0. The graphs show the total number of fusion events (**D**) and the resulting fluorescence changes (**E**) expressed as Area Under Curve (AUC) for each construct. Data are normalized for the cell area and are the mean ± SE of up to 20 cells per construct. *p < 0.05 versus LRRK2 wild-type; °°p < 0.01, °°°p < 0.001 WD40 versus LRRK2 WT or G2385R expressing cell, ANOVA.

Our previous findings suggest that LRRK2 binds and mobilizes SV through a specific set of protein-protein interactions taking place at the WD40 domain^6,29^. Thus we studied the impact on SV trafficking of the G2385R WD40 domain variant. To this aim, we analyzed by TIRFM the trafficking of sypHy vesicles upon over-expression of LRRK2 wild-type or G2385R variant (Fig. 4A). Western-blotting analysis showed that LRRK2-derived constructs are expressed in a similar extent and do not affect significantly sypHy level (Fig. 4B and Supplementary Figure 2A,B). Notably, while single peak fluorescence remained mostly not affected (Supplementary Figure 2E), we reported an increase in the number of fusion events upon LRRK2 G2385R expression (Fig. 4D–F). This result may arise from an increase in the number of sypHy-positive vesicles or instead from a redistribution of vesicles towards the membrane. To discriminate between these two scenarios, we revealed the total pool of sypHy vesicles via incubation with NH_4_Cl_2_. By epifluorescence microscopy we did not find any major difference in total vesicle number between LRRK2 wild-type or G2385 expressing cells (Supplementary Figure 2F,G). Instead, by restricting our analysis to the TIRF zone (i.e within a range of up to 100 nm away from the plasma membrane) we identified significantly more sypHy clusters in cells expressing LRRK2 G2385R (Fig. 4G,H). We have previously shown that LRRK2 acute silencing correlates with an increase in SV fusion^29^. Western-blotting analysis showed that siLRRK2 transfection decreases endogenous LRRK2 level but does not affect significantly sypHy level (Fig. 4C and Supplementary Figure 2C,D). Next we verified the functional impact of LRRK2 down-regulation by TIRFM. Interestingly, we noticed that upon silencing of endogenous LRRK2, cells exhibited an increase in fusion events (Fig. 4D–F) as well as an increase in sypHy clusters within the TIRF zone (Fig. 4G,H). Collectively, these findings suggest that either LRRK2 silencing or G2385R expression increases the number of fusion events by redistributing vesicles near the membrane. To further prove that G2385R variant behaves as a loss-of-function mutation, we tried to rescue its phenotype by co-expressing isolated WD40 domain. We analysed vesicle fusion in stable N2A line expressing either LRRK2 WT or G2385R transfected with sypHy reporter alone or together with RFP-WD40 construct (Fig. 5A). Western-blotting analysis showed that LRRK2-derived constructs are expressed in a similar extent and do not affect significantly sypHy level (Fig. 5B and Supplementary Figure 3A–C). As measured in the SH-SY5Y cell line, the G2385R variant increased the number of fusion events and the associated fluorescence changes with respect to the LRRK2 WT. Overall, while single peak fluorescence remained mostly unaffected (Supplementary Figure 3D), we noticed that the co-expression of isolated WD40 domain abolished the increase in the number of fusion events seen upon LRRK2 G2385R expression (Fig. 5C–E).

### The G2385R PD-risk variant alters LRRK2-binding properties

**Figure 6:**
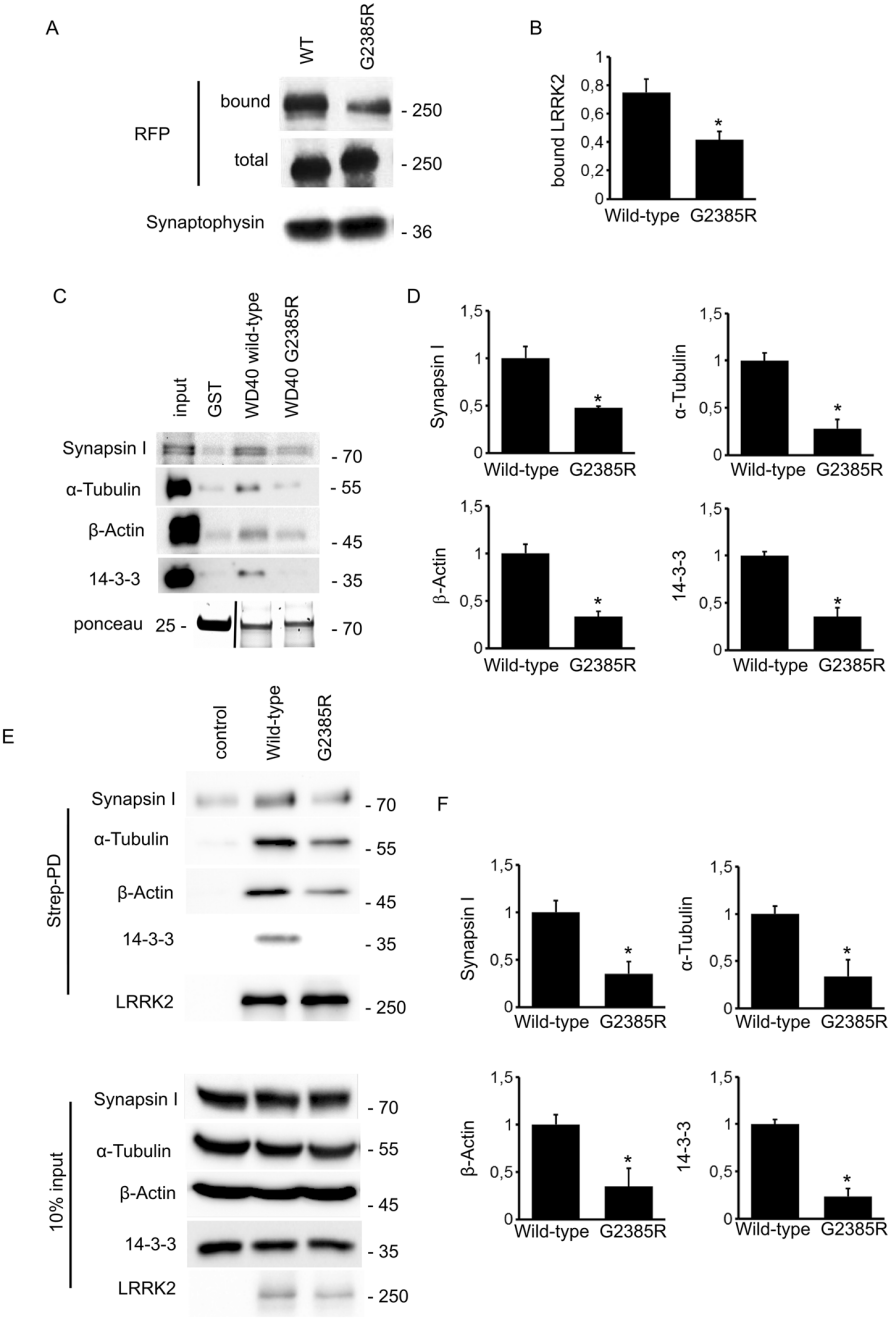
G2385R mutation affects LRRK2 binding properties. (**A**) We measured the extent of LRRK2 and SV binding by ultracentrifugation sedimentation assay. We incubated purified RFP-LRRK2 wild-type and G2385R variant with isolated SV (10 μg protein/sample). Bound RFP-LRRK2 was separated from free RFP-LRRK2 by high-speed centrifugation. We appreciated SV-bound LRRK2 by immunoblotting with anti-RFP antibody. The recovery of SV in the pellet was evaluated based on synaptophysin immunoreactivity. (**B**) The binding of RFP-LRRK2 wild-type and G2385R to SV was calculated as the ratio of total RFP-LRRK2 and expressed as mean ± SE; n = 6. *p < 0.05; Student’s t-test. (**C**) GST-pull down approach was performed to explore the interactome associated to LRRK2 WD40 domain. (Upper panels) GST-fusion proteins corresponding to WD40 domain of LRRK2 wild type and LRRK2 G2385R were used to retain interactors from adult forebrain lysate. (Lower panel) Ponceau staining shows the abundance of GST fusion proteins used as bait. (**D**) We evaluated the extent of synapsin I, α-tubulin, β-Actin and 14-3-3 bound to WD40 G2385R domain. Data are expressed as ratio over WD40 wild-type domain. Graph reports mean ± S.E; n = 4. *p < 0.05, Student’s T-test. (**E**) We isolated on streptavidin resin full-length FLAG-LRRK2 wild-type and FLAG-LRRK2 G2385R protein from N2A over-expressing cells. Interacting proteins were resolved by western-blotting. (**F**) We evaluated the extent of synapsin I, α-tubulin, β-Actin and 14-3-3 bound to the different LRRK2 variant. Data are expressed as the ratio over LRRK2 wild-type. Graphs report mean ± S.E; n = 4. *p < 0.05, Student’s T-test.

The G2385R mutation within LRRK2 WD40 domain has been suggested to interfere with LRRK2 biochemical properties^6,30–32^. We therefore asked whether the G2385R variant impairs LRRK2 interaction with SV. To test this hypothesis, we incubated full-length LRRK2 wild-type or G2385R RFP-fusion protein with purified SV at a nanomolar concentration and tested the extent of binding in a high-speed sedimentation assay. Western blotting analysis using a RFP-specific antibody revealed that LRRK2 G2385R binds less efficiently to purified SV (Fig. 6A,B). The reduced stability of G2385R binding to SV might arise from slight differences between wild type and mutant proteins in terms of affinity/avidity or nature of interactions with SV proteins. In order to address this question we analysed the binding properties of the two LRRK2 WD40 variants coupling a domain-based GST pull down assay to LC-MS/MS. To obtain a relative quantification of the proteins bound to the two domains, we took advantage of mass spectrometry-based label-free quantification, a recent development which allows the identification of specific protein binders^33^. In the initial MS-analysis, 235 proteins were significantly enriched in the GST-WD40 bait condition, compared to the GST-control. Among these proteins, 95 were significantly reduced in the GST-WD40 G2385R condition when compared to the wild-type. Interestingly, among these differentially bound proteins we found cytoskeletal elements such as tubulin and actin as well as various vesicular proteins, including Rabs, synapsin and 14-3-3 (Table 1 and Supplementary Table 1). By western-blotting, we confirmed that GST-WD40 G2385R domain binds to actin, synapsin, tubulin and 14-3-3 with reduced affinity (Fig. 6C,D). To further validate our findings, we investigated the protein complex associated with full-length LRRK2 wild-type and G2385R variants. To this aim, we purified full length Strep-FLAG LRRK2 wild-type and G2385R variants from N2A cell lines. Eluted proteins were resolved by western-blotting. This approach confirmed that G2385R variant reduces LRRK2 affinity towards synapsin, actin, tubulin and 14-3-3 (Fig. 6E,F). Taken together, these results suggest that structural alterations induced by the G2385R substitution functionally modify LRRK2 binding properties thereby diminishing its interaction with a specific subset of vesicular as well as cytoskeletal proteins.

**Table 1:** G2385R variant modifies WD40 binding properties.

Protein names	Protein IDs	Mol. weight [kDa]	G2385R/WT	p Value
Actin-related protein 2/3 complex subunit 3	Q9JM76	20,524	0,23	0,03
Actin-related protein 3	Q99JY9	47,357	0,19	0,01
Actin-related protein 2	P61161	44,76	0,52	0,04
WD repeat-containing protein 1	O88342	66,406	0,34	0,01
Actin, cytoplasmic 2	P63260	41,792	0,37	0,01
Actin-related protein 2/3 complex subunit 1 A	Q9R0Q6	41,626	0,26	0,03
Dihydropyrimidinase-related protein 4	O35098	61,961	0,46	0,01
Ras-related protein Rab-1A	P62821	22,677	0,62	0,03
Guanine deaminase	Q9R111	51,012	0,15	0,01
Dihydropyrimidinase-related protein 1	P97427	62,167	0,42	0,01
Tubulin beta-4A chain	Q9D6F9	49,585	0,39	0,04
Synapsin-2	Q64332	63,372	0,53	0,03
Dihydropyrimidinase-related protein 2	O08553	62,277	0,50	0,03
14-3-3 protein theta	P68254	27,778	0,35	0,05
F-actin-capping protein subunit alpha-2	P47754	32,967	0,50	0,03
14-3-3 protein beta/alpha	Q9CQV8	28,086	0,37	0,02
Coronin-1A	O89053	50,989	0,37	0,05
14-3-3 protein eta	P68510	28,211	0,52	0,03
CaM kinase-like vesicle-associated protein	Q3UHL1	54,819	0,58	0,04
14-3-3 protein epsilon	P62259	29,174	0,37	0,03
Tubulin alpha-1A chain;	P68369	50,135	0,49	0,04
Tubulin alpha-4A chain	P68368	49,924	0,53	0,02
AP-2 complex subunit alpha-1	P17426	107,66	0,44	0,03
Profilin-2	Q9JJV2	15,032	0,37	0,03
Tubulin beta-4B chain	P68372	49,83	0,49	0,03
Tubulin alpha-1B chain	P05213	50,151	0,52	0,04

LC-MS/MS-identification of LRRK2-WD40 interactors. The table reports protein name, UniProtKB protein IDs, protein molecular weight, ratio of abundance of the protein in G2385R versus wild-type bait and relative P value, n = 4 Student T-test.

### G2385R variant mimics activity-driven events

**Figure 7:**
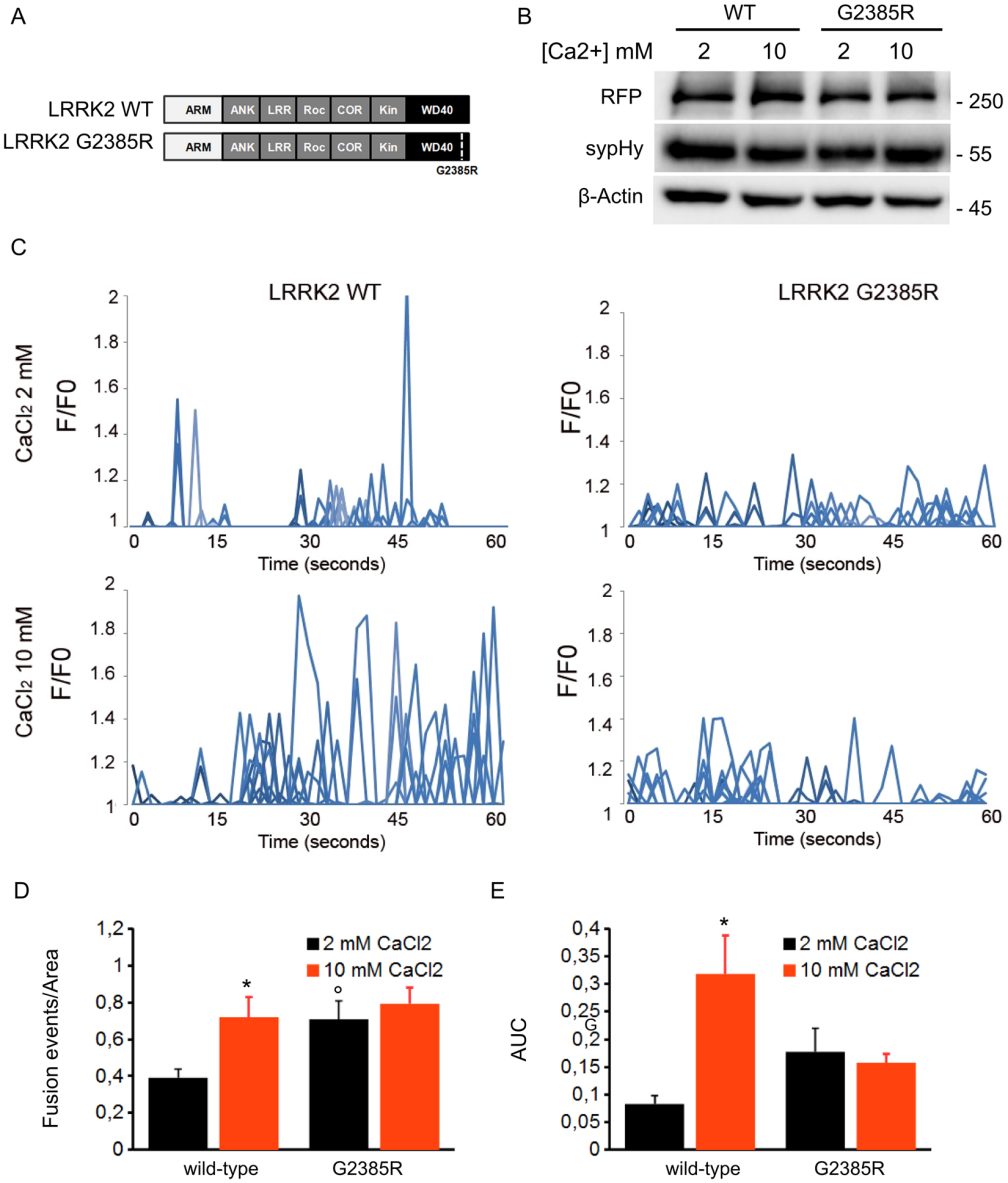
The G2385R variant mimics activity-driven events. (**A**) Schematic representation of LRRK2 wild-type and G2385R variant. The distinct LRRK2 domains are indicated. (**B**) Western-blotting analysis of cells expressing sypHy reporter together with RFP-LRRK2 derived constructs upon incubation with 2 or 10 mM CaCl_2_. (**C**) Time course analysis of synaptic events occurring in SH-5YSY cells cells transfected with wild-type LRRK2 (WT) or G2385R-LRRK2 upon incubation with 2 or 10 mM CaCl_2_ (20 minutes in KRH solution). SH-5YSY cells were co-transfected with sypHy and the indicated constructs and imaged by TIRFM 48 hours later. Peaks of variable fluorescence intensity correspond to single fusion events. Fluorescence data are expressed as F/F0. The graphs show the total number of fusion events (**D**) and the resulting fluorescence changes (**E**) expressed as Area Under Curve (AUC) for each construct. Data are normalized for the cell area and are the mean ± SE of up to 20 cells per construct. *p < 0.05 10 mM versus 2 mM CaCl_2_, °p < 0.05 G2385R versus LRRK2 wild-type, 2 mM CaCl_2_, ANOVA.

Neuronal activity triggers vesicle release via increase in presynaptic calcium^34^. Thus we triggered exocytosis by increasing bath [Ca^2+^] to 10 mM in SH-5Y5Y cells expressing either LRRK2 wild-type or G2385R variant (Fig. 7A). Western-blotting analysis showed that in all the conditions studied, LRRK2-derived constructs and sypHy report are expressed in a similar extent (Fig. 7B and Supplementary Figure 3E,F). As expected, we noticed a significant increase in the number of fusion events, area under the curve (Fig. 7C–E) as well as single peak fluorescence intensity (Supplementary Figure 3G) in LRRK2 wild-type expressing cell upon 10 mM CaCl2 incubation. Instead, we did not observe any additional increase in the number of fusion events and area under the curve in cells expressing LRRK2 G2385R variant. All together these data suggest that G2385R variant mimics activity-driven events.

## Discussion

By applying TIRFM to SH-SY5Y cells expressing synaptopHluorin we dissected the functional implication of the different LRRK2 domains in the context of SV fusion. We noticed a severe impairment of SV fusion events upon over-expression of a construct containing the isolated LRRK2 WD40 domain while, in contrast, the over-expression of LRRK2 variant lacking the WD40 domain increased the number of event. These data are in agreement with our previous finding describing the exo-endocytic activity in neurons expressing isolated WD40 domain or C-terminal truncated LRRK2^6^. Interestingly, LRRK2 binds the β3-Ca_V_ 2.1 channel subunit at N-terminus while synapsin and actin are bound mainly via the WD40 C-terminus [here and^6,23,29^. β3 Ca_V_ 2.1 channel subunit is an essential component of voltage regulated calcium channel and is a master regulator of exocytosis. In fact, CaV2.1 channels allow the activity-driven increase in local Ca^2+^ concentration triggering SV fusion^35,36^. Under resting conditions, SV are tethered by cross-linking via synapsin proteins to the actin cytoskeleton^27,37^. Thus at the presynaptic bouton LRRK2 brings together SV and proteins executing opposite functions: it binds proteins supporting SV fusion (such as β3 Ca_V_ 2.1 channel subunit) as well as those hampering exocytosis (actin and synapsin). Interestingly, we found that LRRK2 interactome is dynamically regulated by activity: in fact, we reported a reduced LRRK2 affinity for synapsin, actin and 14-3-3 upon high calcium stimulation. Our biochemical investigation does not have the necessary temporal and spatial resolution to precisely dissect whether these events take place at the presynaptic bouton upon action potential arrival. However, it is tempting to speculate that LRRK2 acts as a molecular scaffold that integrates neuronal stimuli into defined subsets of protein complexes to modulate SV release. In the basal condition LRRK2 may organize a balanced equilibrium between pro-exocytic and hampering molecules, while, upon stimulation, it may release confining effectors such as actin and synapsin to allow efficient SV fusion. Thus, it is evident that, besides its enzymatic activities, the investigation into the dynamics of the protein complex associated to LRRK2 deserves further studies. Within this context, the C-terminal WD40 domain may play a crucial role. In fact, we have shown that the WD40 domain interacts with SV as well as several presynaptic proteins^6^. Furthermore, a LRRK2 deletion mutant, lacking the entire WD40 domain, fails to induce apoptosis and toxicity concomitant with a lack of kinase activity^30,31^. It is still open whether the kinase domain or WD40 domain or both are causative in this case. The description of the G2385R missense variant within the WD40 domain as the main PD risk variant in the Chinese Han and Korean population gives further functional and pathological relevance to this domain^22^. G2385R carrier patients have clinical signs indistinguishable from idiopathic PD patients. Still, the G2385R variant positively correlates with significant lowering in the age of PD onset^21^. The functional consequences of the G2385R variant remain almost enigmatic so far. It has been shown that this variant has an impact on LRRK2 biochemical properties at different levels such as reduced LRRK2 kinase activity and autophosphorylation *in vitro* ^30^. In silico and electron microscopy-based analyses suggest that the G2385R variant, that replaces a uncharged glycine residue with a charged arginine, induces structural stress via charge-charge repulsion between WD repeats 5 and 6^6^. These changes are predicted to alter the biochemical properties of LRRK2 C-terminal domain. We report here that the G2385R variant affects LRRK2-SV interaction, adding to our previous result on the isolated WD40 domain. By following the “guilt by association” hypothesis^38^ we further investigated the G2385R-specific protein interactome. We demonstrated that the G2385R variant binds less efficiently to some key presynaptic proteins, including actin and synapsin. Thus, the G2385R variant impairs LRRK2 binding to these two major SV tethering elements as well as with SV themselves. Interestingly, we reported an increased number of sypHy-positive vesicles within the TIRFM zone, up to 100 nm away from the cell membrane. In the presynaptic bouton, SV are organized in distinct functional pool, namely ready-releasable pool and resting pool. Ready-releasable SV are those recruited first during synapse activity^39^ and are distributed without a specific localization within the bouton in a range of 100 nm from the active zone^35^. Although the SH-5Y5Y cellular model may not fully recapitulate the entire molecular complex organizing SV trafficking in the presynaptic bouton, we can speculate that the G2385R variant partially abrogates the function of LRRK2 in restraining vesicle movement. An altered binding of LRRK2 to synapsin and actin as well as to SV might allow the redistribution of vesicle towards the membrane and, eventually, promote a more efficient fusion. Accordingly, our data suggest that the G2385R variant mimics biochemically and functionally activity driven events. Finally, we observed that LRRK2 acute silencing and LRRK2 G2385R over-expression have a similar impact on the trafficking and distribution of synaptophluorin-positive vesicles in SH-SY5Y cells. In agreement with previous data from Rudenko and colleagues^30^, this finding suggests that the G2385R variant abolishes the physiological activity of LRRK2 and, in consequence, behaves as a partial loss of function mutation.

## Conclusions

Our findings show that the nature of the macro-molecular complex formed by LRRK2 is tightly correlated to its physiological and pathological function. Furthermore, our data support the idea that the G2385R variant induces a loss of function rather than a gain-of-function phenotype. This suggests that the pathophysiology underlying the dominant nature of LRRK2-associated PD might be more complex and the functional impact of other PD mutations may need reconsideration.

## Methods

### Generation of in-house antibodies

For the generation of the novel anti-LRRK2 antibody, a His-GST fusion protein containing the ankyrin domain (amino acids 688-901) of human LRRK2 was generated by cloning of the corresponding cDNA into the pETM30 vector (provided by the EMBL) and recombinantly expressed in *E. coli*. The soluble protein fraction was purified via the N-terminal 6xHis tag using Ni-NTA Agarose (Qiagen) following standard protocols provided by the manufacturer. Immunization of rats and the generation stable hybridoma were performed in-house (Service Unit Monoclonal Antibodies, Helmholtz-Zentrum München) as described previously^40^. Finally, a rat monoclonal IgG2a clone (LANK/24D8) was established.

### Plasmids and transfection

Human LRRK2 was subcloned into pDEST57 (N-terminal red fluorescent protein RFP tag, Invitrogen) and N-terminal Strep-FLAG (SF-TAP) plasmid^41^ using the Gateway system (Invitrogen). Full-length RFP and Strep-FLAG G2385R variants were generated by site-directed mutagenesis using the QuikChange mutagenesis kit (Stratagene). GST-LRRK2 WD40 wild-type (WT) or GST-LRRK2 WD40 G2385R and RFP LRRK2 1-2141 (hereinafter termed as LRRK2ΔWD40) were described in^6^. LRRK2 silencing and controls shRNA (respectively siLRRK2 and siControl) were previously described^42^. PolIII and shRNA cassette were cut from the pLVTH vector and subcloned in the pSuper vector co-expressing DsRed fluorescent protein via EcoRI and ClaI digestion. SynaptopHluorin expressing vector was previously described^24^. N2A and SH-SY5Y cells were transfected with the different constructs for 48 hours using Lipofectamine 2000 (Invitrogen). Stable N2A clones expressing LRRK2 wild-type or G2385R variant were isolated upon Neomycin selection (1 mg/ml).

### Cell cultures

N2A (Neuro2a, ATCC CCL-131) and SH-SY5Y (ATCC CCL-2266) cells were grown in DMEM with 10% FBS, 1% penicillin/streptomicin and 1% glutamine in a humidified atmosphere of 5% CO_2_ at 37 °C. Cortical neuron cultures were prepared from embryonic day 15.5–16.5 mouse cortexes (C56BL/6 J). Medium-density (150–200 cells/mm^2^) neuron cultures were plated on 12 mm diameter cover-slips in 24-well plastic tissue culture plates (Iwaki; Bibby Sterilin Staffordshire, UK) and grown as described^43^. Cells were processed at DIV14.

### Pull-down and co-immunoprecipitation

LRRK2 GST-fusion domains were expressed in the *E. coli* BL21 strain (Invitrogen), purified as described earlier^6^. Briefly, 5 μg of each GST fusion protein was loaded onto Glutathion-Sepharose Resin (GE-Healthcare, Freiburg) and co-incubated with adult mouse brain lysate or crude SV fraction (LP2, see below) (1 mg of total protein) for 2 h at 4 °C. Beads were washed twice with wash buffer (150 mM NaCl, 2 mM EDTA, 50 mM Tris-HCl and 0.2% Triton, pH 7.4) and interacting proteins were eluted in Sample Buffer 2X for 10 min at 55 °C. For LRRK2 pull-down from N2A cells, 48 h after transfection cells were solubilized in lysis buffer (150 mM NaCl, 2 mM EDTA, 50 mM Tris-HCl, 1% NP-40 and 0.25% sodium deoxycholate, pH 7.4) with protease and phosphatase inhibitors (Calbiochem) for 1 h at 4 °C. LRRK2 was precipitated using Strep-Tactin Superflow resin (Iba) for 2 h at 4 °C. Washing conditions were performed with high salt buffer (300 mM NaCl, 50 mM Tris-HCl pH 7.4). Interacting proteins were eluted in Laemmli buffer 2× at 55 °C for 10 minutes. In order to purify RFP-LRRK2 WT and G2385R, cells were processed with RFP-Trap_A kit according to manufacturer’s protocol. For endogenous LRRK2 immunoprecipitation, adult mouse forebrain or DIV14 cortical cultures were solubilized in lysis buffer (150 mM NaCl, 2 mM EDTA, 50 mM Tris-HCl, 1% NP-40 and 0.25% sodium deoxycholate, pH 7.4), protease and phosphatase inhibitors cocktail (Calbiochem) by mechanical disaggregation. Where indicated neuronal cultures were incubated in medium containing 2 mM or 10 mM CaCl_2_. Protein amount was quantified according to Bradford’s method and 1 mg of total protein was incubated with 4 µg of rat anti-LRRK2 (LANK/24D8 clone) or rat anti-IgG (Abcam). Brain homogenates were pre-cleared on sepharose beads (GE-Healthcare, Freiburg, Germany). Immunocomplexes were precipitated using protein G-Sepharose beads (GE-Healthcare, Freiburg, Germany) and proteins bound to LRRK2 were extracted in Laemmli buffer 2× at 55 °C for 10 minutes. For protein identification by Western blotting, samples were loaded onto a 10% SDS-PAGE gel and transferred into nitrocellulose membrane (Amersham) at 82 V for 2 h at 4 °C. The primary antibodies were applied overnight at 4 °C in blocking buffer (20 mM Tris, pH 7.4, 150 mM NaCl, 0.1% Tween 20, and 5% non-fat dry milk); primary antibodies included: rabbit anti-LRRK2 1:500 (MJFF2, c41-2), rabbit anti-RFP 1:250 (Abcam), rabbit anti- β3-Cav2.1 1:200, mouse anti-β-Actin 1:4000, mouse anti-α-tubulin 1:2000, mouse anti-syntaxin 1 1:1000 (Sigma), rabbit anti-Synapsin I 1:1000, rabbit anti-NSF 1:1000 (Cell Signalling), rabbit anti-pan-14-3-3 1:1000 (Santa Cruz). The secondary antibodies HRP-conjugated anti rabbit or anti mouse (Jackson Immunoresearch) were used at 1:7000 dilution. Proteins were detected using the ECL prime detection system (GE Healthcare) taking advantage of ChemiDoc Touch Imaging system (Bio-rad). Band intensity was quantified on ImageJ. To appreciate co-immunoprecipitation efficiency, we normalized the intensity of the co-immunoprecipitated protein to the amount of LRRK2 variant immunoprecipitated. Pull-down efficiency was judged as amount of preys normalized to bait quantity as monitored by Ponceau staining.

### Immunofluorescence staining

Neurons were fixed at DIV 14 in cold methanol 100% for 10 min. Primary antibodies were applied in GDB buffer (30 mM phosphate buffer, pH 7.4, containing 0.2% gelatin, 0.5% Triton X-100, and 0.8 M NaCl), overnight at 4 °C. Primary antibodies used were: mouse anti-LRRK2 1:100 (clone N231B/34, NeuroMab), rabbit anti-β-3Cav2.1 1:50 (Sigma), rabbit anti-synapsin I 1:200 (Cell Signalling). Secondary antibodies were prepared in GDB buffer and used for 2 h at room temperature; secondary antibodies include: goat anti-mouse Alexa Fluor 488 (Invitrogen), goat anti-rabbit Cy3 (Jackson Immunoresearch) and Alexa Fluor 555 Phalloidin (Molecular Probes, Life Technologies). Cover slips were mounted with prolonged reagent (Life Technologies) and observed with Zeiss Observer Z1 microscope equipped with an Apotome module. The obtained images provide an axial resolution comparable to confocal microscopy^44,45^. Images were acquired with AxioObserv Z1 microscope equipped with Apotome module using a plan-Apochromat 63×/1.40 Oil objective, pixel size 0,102 μm × 0.102 μm. Colocalization studies were performed on the single plan generated by optical sectioning elaborated by Apotome module. Acquired images were analyzed with ImageJ software using the colocalization analysis plugin to detect colocalization between LRRK2 and interactors.

### Synaptic vesicle isolation and binding assay

Highly purified rat SV were obtained through controlled-pore glass chromatography^46^. The binding of LRRK2 fusion proteins to SV was carried out using a high-speed sedimentation assay^47^. Briefly, SV (5–10 µmg total protein) were incubated for 1 hour at 0 °C with RFP-LRRK2 WT or RFP-LRRK2 G2385R protein in a buffer containing 220 mM glycine, 30 mM NaCl, 5 mM Tris/HCl, 4 mM Hepes (pH 7.4), 0.22 mM NaN_3_, 0.2 mM PMSF, 2 μg/ml pepstatin and 100 µg/mL of bovine serum albumin (BSA). After the incubation, LRRK2 bound to SV were separated by high-speed centrifugation (400,000 × g for 45 min). Aliquots of the resuspended pellets were subjected to SDS–PAGE and subsequent Western blotting with LRRK2-specific antibodies. The amount of LRRK2 was determined in function of optical density in comparison to known amount of fusion proteins. The recovery of SV, used to correct the amounts of LRRK2 bound to SV, was determined by Western blotting with anti-synaptophysin antibodies.

### MS/MS identification

The mass spectrometric analysis was performed as described previously^6^. Each replicate was analyzed as a single run by LC-MSMS using a nano-flow HPLC system (Dionex Ultimate 3000 RSLC, Thermo Scientific) coupled to a Orbitrap XL mass spectrometer (Thermo-Scientific). The Raw data were directly analyzed by the MaxQuant Software ver. 1.5.2.8 (www.maxquant.org) allowing protein identification and label-free quantification^48^ with the following Andomeda search-engine parameters: database: Swissprot database mouse subset (v2016-09, 16,811 entries), *E. coli* K12 subset (v2016-09, 4,252 entries), sequence of the bait (LRRK2) and fusion tag (GST). Carbamidomethylation was set as fixed modification as well as N-terminal protein acetylation as variable modification. As enzyme trypsin was set. The initial mass accuracy (MS) for mass recalibration was set to 20 ppm. For MS/MS spectra, the mass accuracy was set to 0.1 Da. The FDR (false discovery rate) threshold was set to 0.01. Razor-peptides and peptides with N-terminal acetylation or methionine oxidation were included into protein quantification. Only proteins identified by at least two peptides were quantified. Downstream label-free quantification and statistical analysis was carried out following established workflows in Perseus ver. 1.5.4.0 as described in^33^. Briefly, for the analysis, 4 independent samples per condition (GST-control, GST-WD40 and GST-WD40-G2385R) have been considered. Only IDs with at least 3 appearances in one condition have been included. Samples have been normalized by the label free-quantification (LFQ) values corresponding to the GST-tag. Missing values have been replaced by the build-in imputation function of Perseus. Specific interactors for the WD40 domain have been determined by the built-in two sample test function (Student’s T-test), followed by a permutation-based FDR (FDR = 0.05, number of randomerizations = 250). For these interactors a comparison of WT v.s. mutant has been performed and the statistical significance has been determined with the same test as described above.

### Time lapse microscopy by Total internal reflection fluorescence microscopy (TIRFM)

48 hours after transfection, transfected cells were imaged through a TIRF microscope (Carl Zeiss Inc.) equipped with an Argon laser at 25 °C using a 100 × 1.45 numerical aperture (NA) oil immersion objective as in^25^. Green fluorescence was excited using the 488-nm laser line and imaged through a band-pass filter (Zeiss) onto a Retiga SRV CCD camera. Single-cell imaging under TIRF illumination was carried out at 1 hz for a total of one minute, in a standard saline solution (KRH) containing in mM: 125 NaCl, 5 KCl, 2 CaCl2, 1.2 MgSO4, 1.2 KH2PO4, 5 Glucose, 25 4-(2-Hydroxyethyl)-piperazine-1-ethanesulfonic acid (HEPES) (buffered to pH 7.4) at room temperature (25 °C). Where indicated, cells were incubated in a KRH solution containing 10 mM [CaCl2] for 20 minutes, before recording. Up to ten cells were imaged on each coverslip in at least three independent experiments for each construct. TIRF images were analyzed using Image-Pro Plus Analyser Image Software (Media Cybernetics, Bethesda, MD, USA). A set of automated image processing macro/subroutines was developed on the basis of existing algorithms of the Image-Pro Plus Analyser software (High Pass Gaussian filtering, nearest neighbouring deconvolution). Finally, the images were background subtracted by using the averaged value within a user-defined background region and corrected for photobleaching. The resulting corrected images were then analyzed using an existing Image-Pro Plus plug-in (tracking object) for selection and quantification of fluorescent spots according to their shapes, size and intensity. The following criteria were used to include individual structures in the analysis: (1) mean area 0.039–1 μm^2^, (2) minimal pixel density 20, (3) aspect (major/minor axis) 1–3, (4) velocity limit search radius 1 (micron/frame). Fixed spots (spots present in the same position in at least 20 frames of the movie) were automatically excluded from the analysis. The obtained data were exported in Excel and further analysed. The fluorescence intensity (F) of each spot in the various frames was normalized to its respective initial intensity (F0) and plotted against time. The number of fusion events and the whole cell fluorescence changes were automatically evaluated by a custom written macro. Data, normalized to the cell area, come from at least 15 cells for each construct. To quantify the vesicle density in the TIRF zone, the cells were incubated for 5 minutes with 50 mM NH_4_Cl_2_ in KRH solution, to label all synapto-pHluorin positive vesicles. Then, cells were fixed in paraformaldehyde and imaged by TIRFM or epifluorescence. The number of vesicles was quantified as described above.

### Statistical analysis and guidelines

All data are expressed as mean ± standard error of the mean (SE). Data were analyzed with an unpaired Student’s t test (two classes) or ANOVA followed by Tuckey’s post-hoc test (more than two classes). The indication of number of experiment (n) and level of significance (p) are indicated throughout the text. All methods were performed in accordance with the relevant guidelines and national regulations. All procedures involving animals were approved by Institutional and National Agencies (autorizzazione 793/2016-PR).

## Electronic supplementary material

supplementary figures 1-3

supplementary table 1
