# Supplementary material for: The LRRK2 G2385R variant is a partial loss-of-function mutation that affects synaptic vesicle trafficking through altered protein interactions
Source: Sci Rep. 2017 Jul 14;7:5377. doi: 10.1038/s41598-017-05760-9 (PMC5511190; doi:10.1038/s41598-017-05760-9)
Supplement: Supplementary file 1 — supplementary figures 1-3 [file 41598_2017_5760_MOESM1_ESM.pdf]

The LRRK2 G2385R variant is a partial loss-of-function mutation that affects synaptic vesicle trafficking through altered protein interactions.

Maria Dolores Perez Carrion, Silvia Marsicano, Federica Daniele, Antonella Marte, Francesca Pischedda, Eliana Di Cairano, Ester Piovesana, Felix von Zweydorf, Elisabeth Kremmer, Christian Johannes Gloeckner, Franco Onofri, Carla Perego*, Giovanni Piccoli*

Supplementary Table 1. Label free quantification of GST pulldown from mouse brain lysates using MaxQuant and Pereus. All hits reported in the table are significantly enriched in the GST-WD40 wild type sample over the GST control. Hits showing a significant difference in enrichment between GST-WD40 G2385R and GST-WD40 wild type are indicated by an asterix (column I). Detailed description: Column A: gene names of hits significantly enriched in GST-WD40 over GST-control. Column B: full protein names. Column C: Uniprot Identifier. Column D: Majority protein IDs as reported by MaxQuant considering protein inference. Column E: Ratio GST-WD40/GST-control. Column F: CRAPome data number of times a protein ID was identified in a total number of 411 experiments reported in the CRAPome database (www.crapome.org). Column G: CRAPome data, as given in column F, in percent. Column G: Ratio GST-WD40 G2385R/GST-WD40 wild-type. Column I: indication of hits showing significant differences in interaction between G2385R and WT. Column J-U: LFQ intensities for four independent experiments with GST-control, GST-WD40 G2385R and GST-WD40 wildtype considered for the statistical analysis. Column V: Molecular weight of the identified protein. Column W: Identification score (Andromeda search engine implemented in MaqQuant). Column X-AI: Number of peptides considering protein inference (Razor+ unique peptides) as reported by MaxQuant separately for each experiment. Column AJ-AU: Number of unique (proteatypic) peptides as reported by MaxQuant separately for each experiment. Column AV-BG: Sequence coverage in [%] as reported by MaxQuant separately for each experiment. Column BH: -log T-test p-values for GST-WD40 compared to GST control as given by Perseus. Column BI: T-test q-values for GST-WD40 compared to GST control as given by Perseus. Column BJ: T-test Difference for GST-WD40 compared to GST control as given by Perseus. Column BK: T-test test statistics GST-WD40 compared to GST control as given by Perseus. Column BL: -log T-test p-values for GST-WD40 G2385R compared to GST-WD40 wild-type as given by Perseus. Column BM: T-test q-values for GST-WD40 G2385R compared to GST-WD40 wildtype as given by Perseus. Column BN: T-test Difference for GST-WD40 G2385R compared to GST-WD40 wild-type as given by Perseus.

Column BO: T-test test statistics GST-WD40 G2385R compared to GST-WD40 wild-type as given by Perseus. Column BP: Median LFQ for GST control. Column BQ: Median LFQ for GST-WD40 G2385R. Column BR: Median LFQ for GST-WD40 wild-type.

Supplementary Figure 1. Expression of LRRK2 and synaptic vesicle proteins in SH-SY5Y cell line**.** (A) Representative western blots showing the expression of LRRK2 and synaptic proteins in SH-5YSY cell line. (B). Representative SH-5YSY cell expressing synaptopHluorin (sypHy) double stained with GFP (green) and VAMP2 (red), visualized under TIRF microscopy. The colocalization between sypHy and VAMP2 is shown in yellow in the merge panel. Scale bar: 10 µm. Inset: 2.5X magnification of a particular region. (C-D) The graphs show RFP (C) and sypHy (D) optical density normalized upon β-actin level as detected by western-blotting. Data are expressed as mean± S.E n=5. (E) The graph shows the average peak height. Data are expressed as mean± S.E. of up to 20 cells per construct

Supplementary Figure 2. (A-D) The graphs show RFP (A), sypHy (B and D) and LRRK2 (C) optical density normalized upon β-actin level as detected by western-blotting. Data are expressed as mean± S.E; n=5. (E) The graph shows the average peak height. Data are expressed as mean± S.E. of up to 15 cells per construct. (F) Vesicles density in transfected SH-5YSY after NH_4Cl_2 treatment. The cells were incubated with the membrane permeable NH_4Cl_2 solution for 5 minutes to label all sypHy positive clusters. Then, cells were fixed and imaged by epifluorescence to count sypHy-positive clusters in the whole cell. Scale bar= 10 µm. (G) The graph reports the number of sypHy positive clusters visualized by epifluorescence. Data are normalized for the cell area and are expressed as mean ± SE; n= 20 cells for each construct.

Supplementary Figure 3. (A-C) The graphs show RFP-LRRK2 (A), sypHy (B) and RFP-WD40 (C) optical density normalized upon β-actin level as detected by western-blotting. Data are expressed as mean ± S.E; n=5. (D) The graph shows the average peak height. Data are expressed as mean± S.E. of up to 20 cells per construct. (E-F) The graphs show RFP-LRRK2 (E) and sypHy (F) optical density normalized upon β-actin level as detected by western-blotting. Data are expressed as mean ± S.E; n=5. (G) The graph shows average peak height. Data are expressed as mean± S.E. of up to 20 cells per construct. * $p<0.05$ versus 2 mM $CaCl_2$, ANOVA.

Supplementary Figure 4. LRRK2 interacts with proteins involved in vesicle trafficking. The original blots relative to the panels included in Figure 2A and Figure 2D are shown.

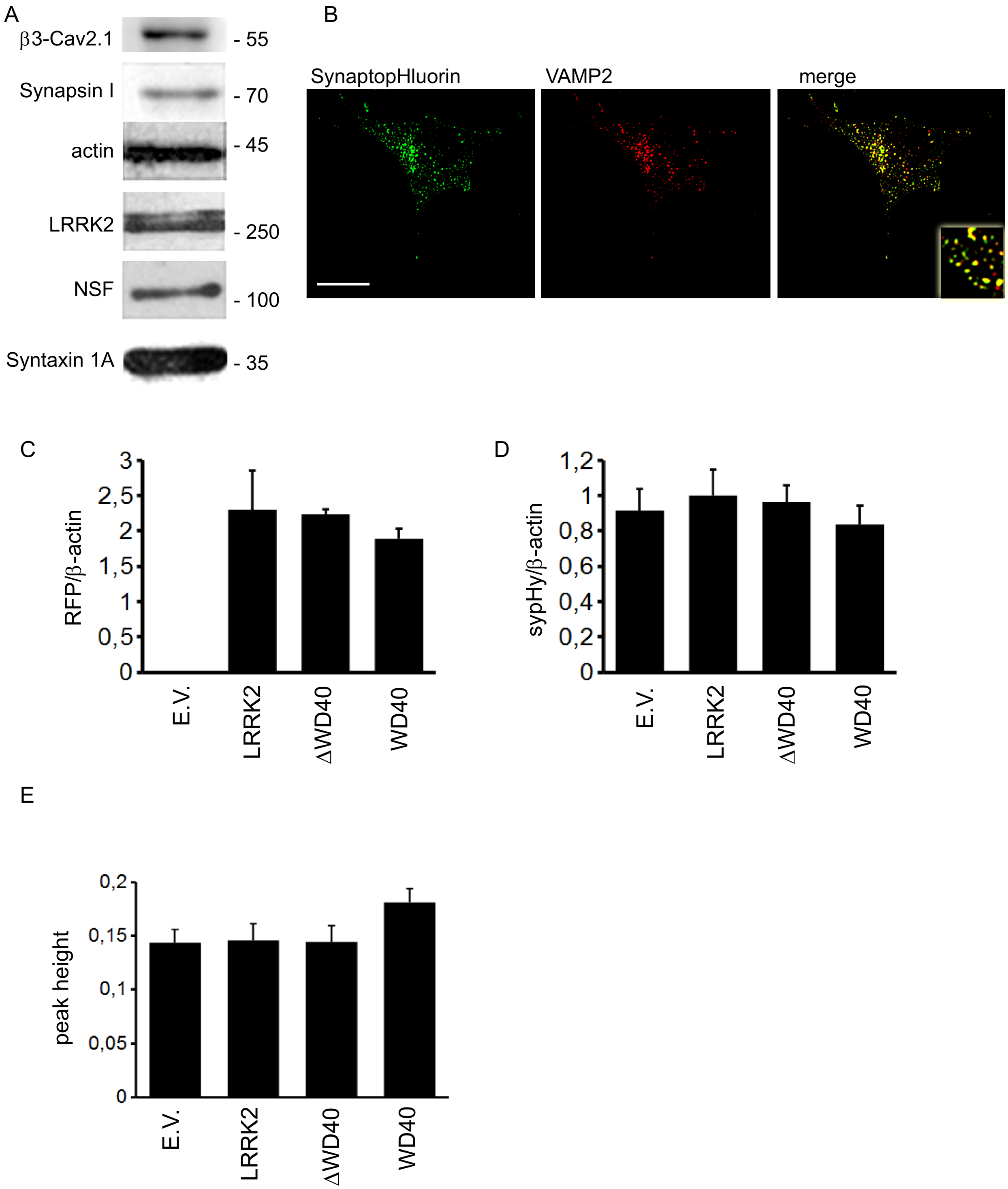

Supplementary Figure 1

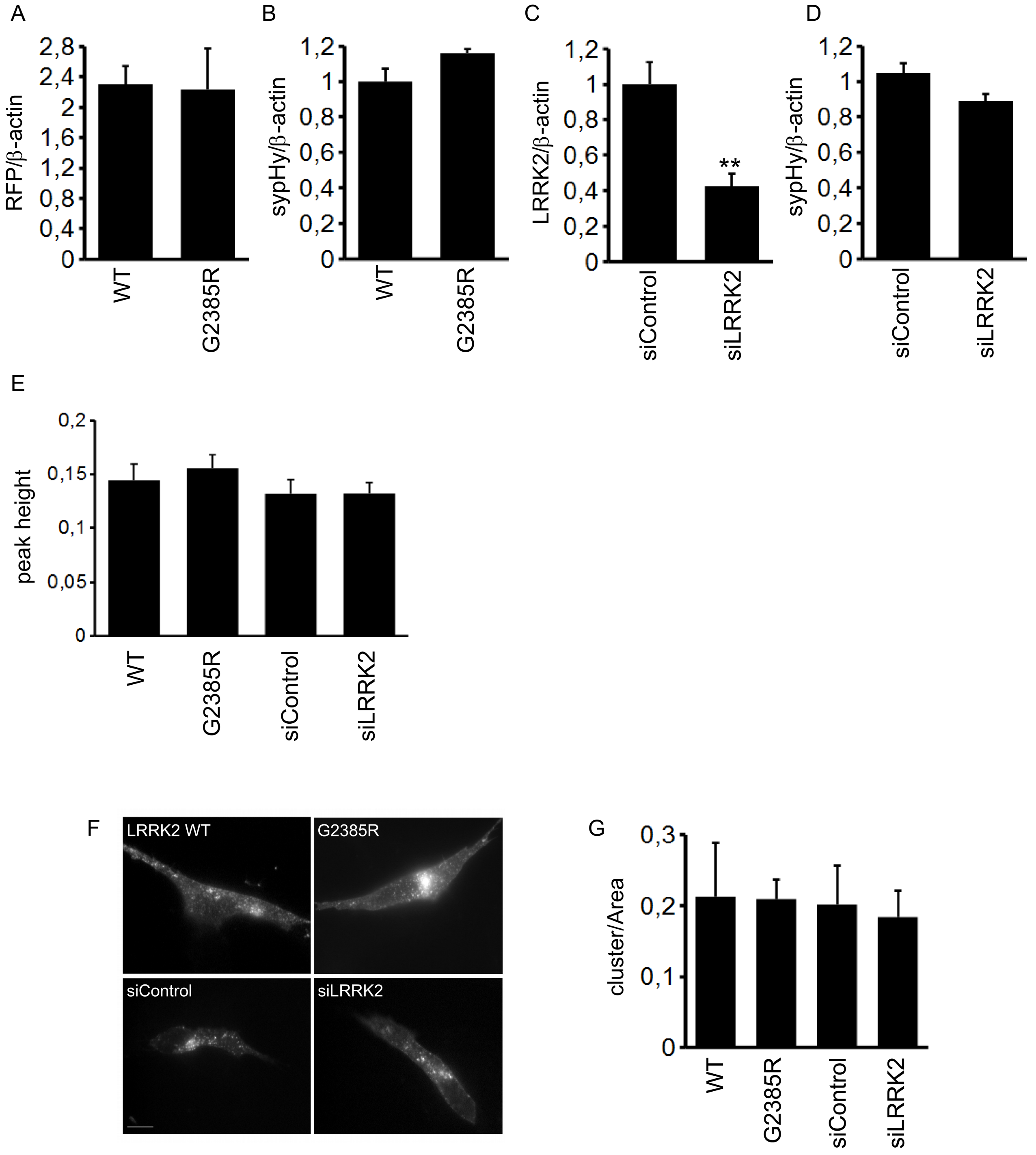

Supplementary Figure 2

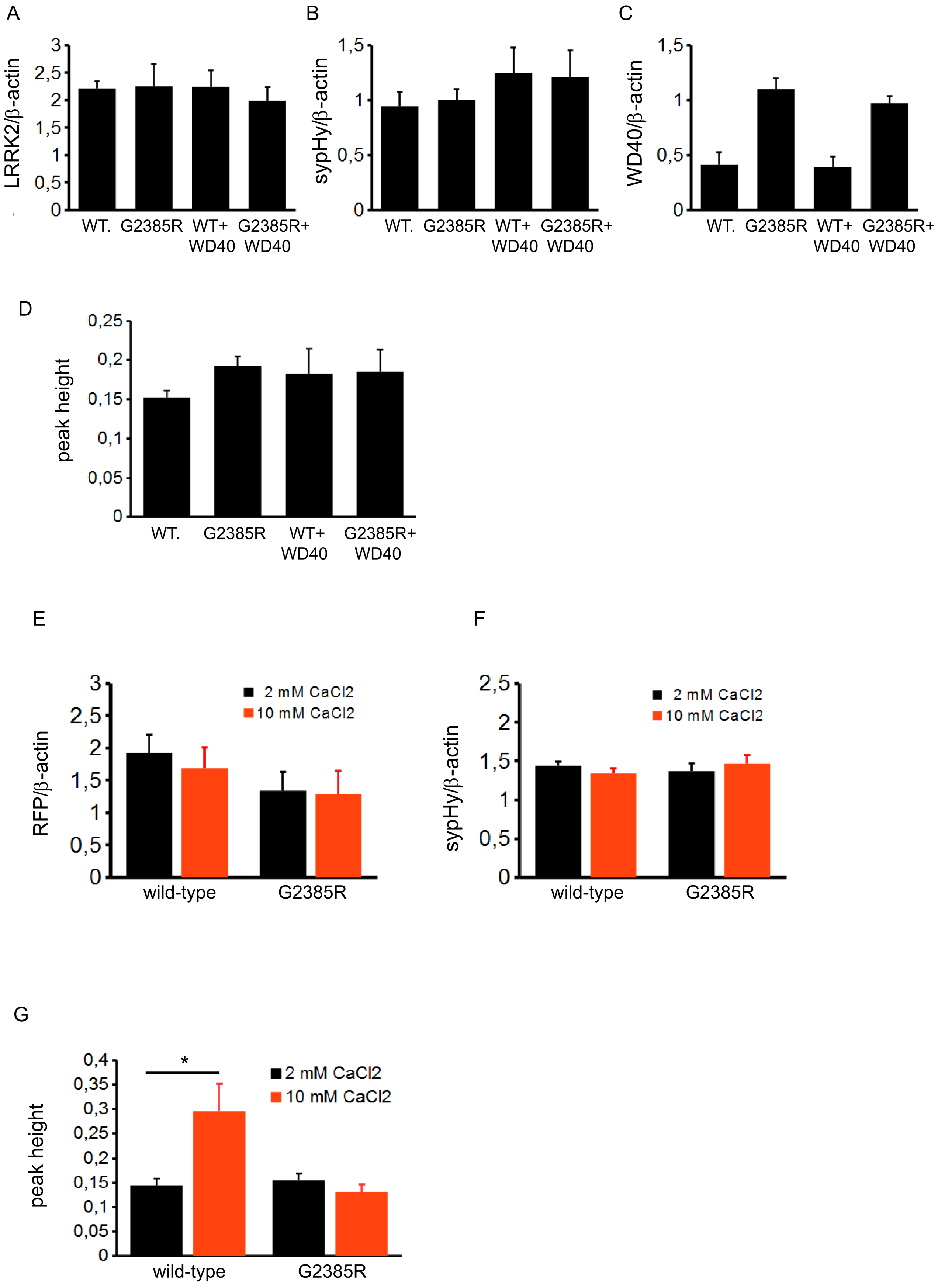

Supplementary Figure 3

Figure 2A

LRRK2

synaptotagmin

actin

synapsin I

β3-Cav2.1

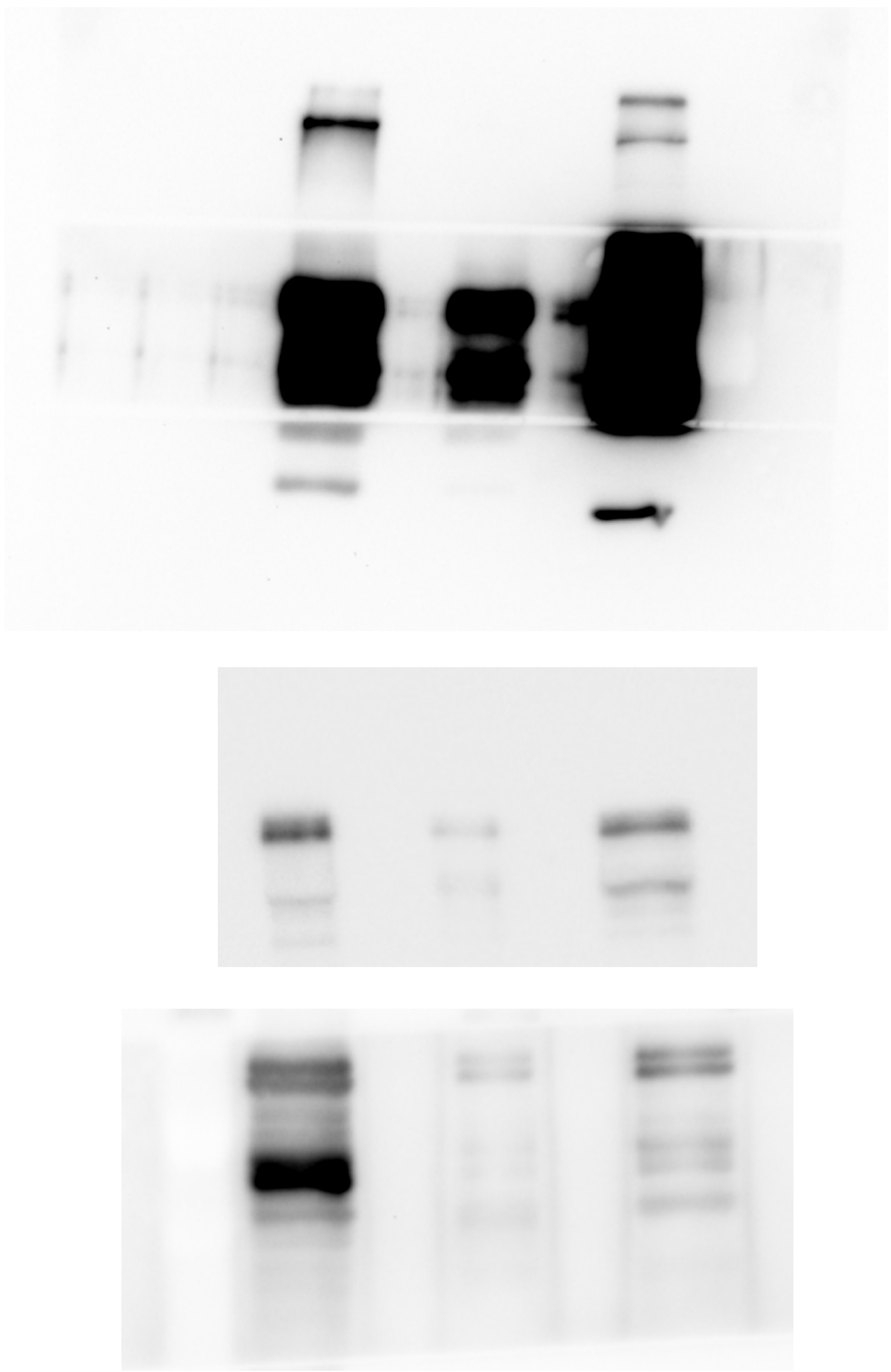

- 250
- 70
- 45
- 70
- 55

Figure 2D

LRRK2

synapsin I

β3-Cav2.1

actin

actin

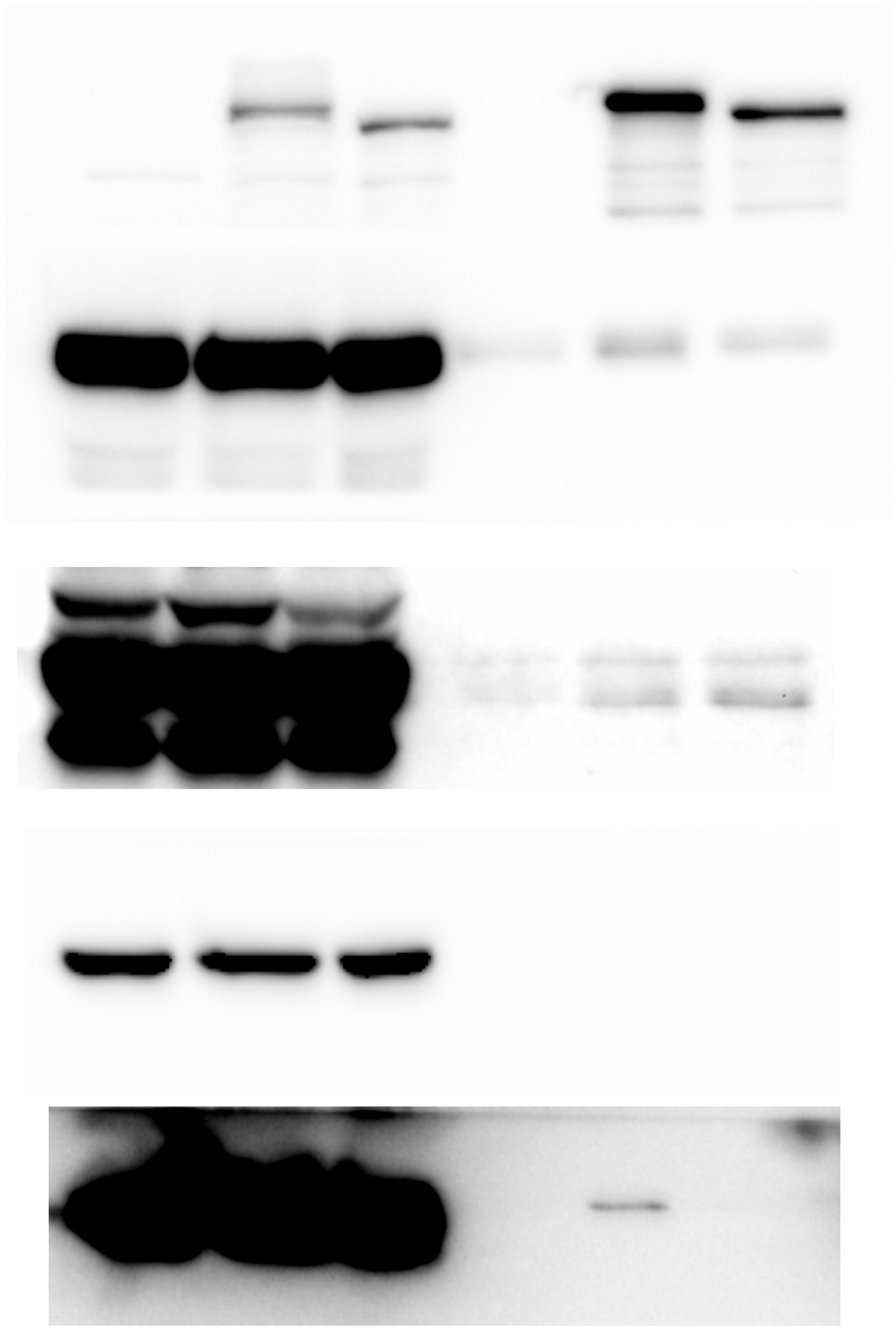

- 250
- 70
- 55
- 45
- 45

(high exposure)

Supplementary figure 4
